# Fibrinolytic niche is required for alveolar type 2 cell-mediated alveologenesis via a uPA-A6-CD44^+^-ENaC signal cascade

**DOI:** 10.1038/s41392-021-00511-9

**Published:** 2021-02-27

**Authors:** Gibran Ali, Mo Zhang, Runzhen Zhao, Krishan G. Jain, Jianjun Chang, Satoshi Komatsu, Beiyun Zhou, Jiurong Liang, Michael A. Matthay, Hong-Long Ji

**Affiliations:** 1 Department of Cellular and Molecular Biology, University of Texas Health Science Center at Tyler, Tyler, TX USA; 2 Institute of Lung and Molecular Therapy, Xinxiang Medical University, Xinxiang, Henan China; 3 Institute of Health Sciences, China Medical University, Shenyang, Liaoning China; 4 Division of Pulmonary, Critical Care and Sleep Medicine, University of Southern California, Los Angeles, CA USA; 5 Hastings Center for Pulmonary Research, University of Southern California, Los Angeles, CA USA; 6 Norris Comprehensive Cancer Center, University of Southern California, Los Angeles, CA USA; 7 Department of Medicine, Keck School of Medicine, University of Southern California, Los Angeles, CA USA; 8 Department of Medicine, Cedars-Sinai Medical Center, Los Angeles, CA USA; 9 Department of Medicine and Anesthesia, University of California San Francisco, San Francisco, CA USA; 10 Cardiovascular Research Institute, University of California San Francisco, San Francisco, CA USA; 11 Texas Lung Injury Institute, University of Texas Health Science Center at Tyler, Tyler, TX USA

**Keywords:** Experimental models of disease, Molecular medicine

**Dear Editor**,

COVID-19, SARS, and MERS are featured by suppressed lung fibrinolysis. The primary objective of this study was to decipher the role of the fibrinolytic niche in the progenitor alveolar type 2 (AT2) cell-mediated re-alveolarization. The expression of *Plau* in AT2 cells has been confirmed at the mRNA, protein, and functional level across species. We employed influenza-infected & *Plau*^*−/−*^ mice, 3D organoids, and polarized monolayers to track AT2 fate. We found a marked reduction in total AT2 cells and CD44^+^ subpopulation when the fibrinolytic niche was disrupted.

The lungs infected with H1N1/PR8 influenza virus displayed widespread alveolar collapse and increased thickness of the lung interstitial septa in a severity-dependent manner. The severity of injured regions was classified as mild, moderate, and severe based on lung injury score (Fig. 1a). Randomly selected fields were analyzed for proSPC^+^ AT2 cells, showing wt two-fold (12.6 ± 0.5%) that of *Plau*^*−/−*^ (6.0 ± 0.3%) mice. AT2 cells/field were significantly reduced in both moderate and severe injury regions vs wt controls. AT2 cells in infected *Plau*^*−/−*^ mice were significantly fewer than in wt controls. This is consistent with a reduction in the in vivo AT2 yield (wt 1.7 × 10^6^ and *Plau*^*−/−*^ 2.6 × 10^6^ cells), suggesting the regulation of AT2 lineage by the fibrinolytic niche for AT2 renewal and differentiation. The viability and purity of AT2 cell isolations was examined as previously described.^1^

**Fig. 1:**
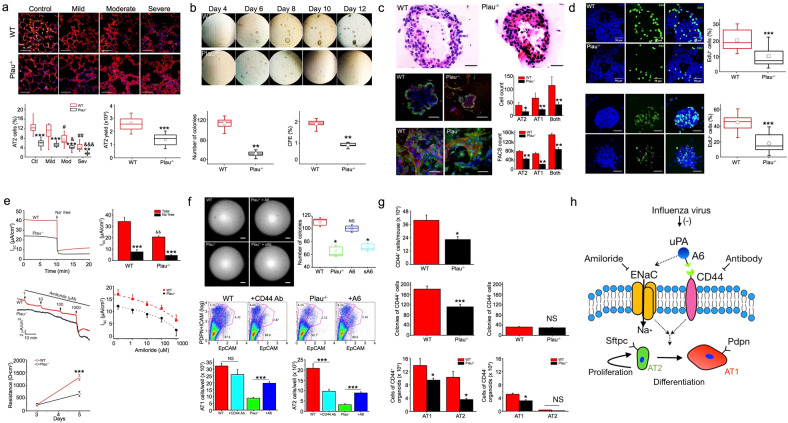
**a** Quantification of AT2 cells in influenza-infected mouse lungs 5 days post infection. *n* = 10 sections from 3 different mice per group. Yield of AT2 cells from *Plau*^−*/−*^ and wt control mice. (*n* = 15 mice/genotype). **b** Colony formation observed and analyzed from day 4 to day 12 post seeding. Colony number and colony forming efficiency (CFE) on day 8 of the cultures. (*n* = 16 wells per set of experiments and repeated with 6 pairs of mice). **c** Representative images of organoid sections stained with the H & E procedure for wt and *Plau*^−*/−*^ group. Stacked confocal images of wt and *Plau*^−*/−*^ organoids. AT1 and AT2 cells were identified and counted as pdpn-positive (red) and sftpc-positive (green) cells, respectively (*n* = 16 organoids per group from three separate experiments). Confocal images and FACS analysis of polarized AT2 monolayers (*n* = 19 transwells per group from three separate experiments). **d** A Click-iT EdU assay kit was used to identify AT2 cells with active DNA synthesis on day 7 of the monolayer cultures (*n* = 16 per group from 6 separate preparations). Stacked organoid images of DAPI for nuclei (left), EdU (middle), and merged (right) in wt and *Plau*^−*/−*^ organoids (*n* = 25 for each group from 6 separate preparations). **e** Representative short-circuit current (Isc) in the presence and absence of bath Na^+^ ions. The bath solution was replaced with Na^+^ free medium as indicated by arrows, summarized Isc level, Isc traces in the presence of accumulating amiloride added to the apical compartment, dotted and dashed lines are generated by fitting raw data points with the Hill equation to compute IC_50_ value for amiloride (130 ± 2.5 μM for wt and 108 ± 14.9 μM for *Plau*^−*/−*^ cells), transepithelial resistance of AT2 monolayers measured with a chopstick meter (*n* = 15 monolayers from 3 different cell preparations). **f** Effects of the A6 peptide and CD44 receptor on the formation of organoids and AT2 lineage in organoids. (*n* = 16 transwells/group from 6 independent experiments). Colony number on day 8 of the cultures. (*n* = 16 wells per set of experiments and repeated with 6 pairs of mice). FlowJo analyzed FACS results. Wt AT2 cells were treated with blocking antibody against CD44 receptor, and *Plau*^−*/−*^ cells were incubated with A6 peptide. Pdpn and EpCAM are biomarkers for AT1 and AT2 cells, respectively. (*n* = 12 transwells for each group from 6 independent experiments). **g** Comparison of the number of EpCAM^+^CD44^+^ AT2 cells between wt and *Plau*^−*/−*^ mice. *n* = 7 mice/genotype. Number of organoids per transwell for CD44^+^ and CD44^−^ AT2 cells. *n* = 18 for each group from four independent experiments. Statistical comparison of FACS data for AT1 and AT2 cells in organoids grown from CD44^+^ and CD44^–^ subpopulations. *n* = 12 transwells per group from three independent experiments. **h** Summary of the uPA-A6-CD44^+^-ENaC cascade in regulating the fate of AT2 cells critical for re-alveologenesis. Scale bar, 50 and 100 µm. Data are means ± sem and analyzed with the Student’s *t* test, pair signed rank test, and one-way ANOVA followed by Tukey post hoc analysis in NS, not significant, *P ≤ 0.05, **P ≤ 0.01, and ***P ≤ 0.001 compared with wt controls

To investigate whether *Plau* gene is required for re-alveolarization, we quantitated AT2 spheroids (Fig. 1b). Organoids with a diameter >50 μm and colony forming efficiency (CFE) were significantly decreased (*n* = 12, *P* < 0.01) in *Plau*^*−/−*^ cultures vs wt controls (Supplementary Fig. 1), apparently, due to a reduction in the organoids with a diameter of 50–200 μm. The suppression in organoid formation associated with *Plau*^*−/−*^ cells contributed to the reduction of total surface area, a clinical parameter for epithelial repair and development. The large organoids were filled with culture medium, having a smaller lumen with thicker walls for *Plau*^*−/−*^ cultures over wt controls (Fig. 1c). These data provide direct evidence for the regulation of AT2-mediated re-alveologenesis by uPA.

Similarly, polarized monolayers exhibited a decline in both AT1 and AT2 cells in *Plau*^*−/−*^ monolayers in the absence of fibroblasts, the feeder cells in organoids. Further, these observations were confirmed by FACS assays. The similarity between organoids and monolayers excludes the potential contribution of fibroblast-released uPA. Cells with active DNA synthesis were assessed by the EdU incorporation assays, showing significantly lesser EdU^+^ cells in *Plau*^*−/−*^ group than in wt controls (Fig. 1d). These results suggest an impaired AT2 self-renewal and potential differentiation into AT1 cells (Fig. 1h).

The divergent bioelectric features in polarized AT2 monolayers, including transepithelial resistance (*R*_T_) and short-circuit currents (*I*_SC_) were measured. *Plau*^*−/−*^ monolayers showed a lower *I*_SC_ level compared to that in wt and diminished significantly by replacing Na^+^-free bath solution to inhibit Na^+^ ion transport, consistent with our previous studies in the airway epithelial cells and that ~20% of apical conductance was inhibited in rat fetal alveolar cells. *Plau*^*−/−*^ cells showed a reduced amiloride-sensitive *I*_SC_ level compared to that in wt groups. Additionally, a greater *R*_TE_ value was recorded in wt than in *Plau*^*−/−*^ monolayers (Fig. 1e). Thus, the ENaC activity seems to be regulated by the *Plau* gene.

CD44 receptors govern cell survival and the progression of lung fibrosis via the Toll-like receptors and hyaluronan.^2^ The connective domain of uPA has an A6 peptide. We demonstrate a new mechanism that CD44 receptors are key players for AT2-mediated re-alveolarization. This is supported by our observations that CD44^+^ AT2 cells have an enhanced capacity for self-renewal and differentiation to AT1 cells. Of note, *Plau*^*−/−*^ mice have shown lesser CD44^+^ AT2 cells compared to those in wt controls. The A6 peptide may serve as a mediator for uPA to bind with CD44 receptors, as A6 peptides restored the dysfunctional fate of *Plau*^*−/−*^ AT2 cells (Fig. 1f). Also, A6 peptide markedly augmented the surface area of *Plau*^*−/−*^ organoids (1.35 ± 0.13 mm^2^/well vs. 0.82 ± 0.05 mm^2^/well). Given that amiloride is a specific inhibitor of urokinase and kills proliferating cells, we could not test its effects on AT2 fate. Parallelly, blockade of CD44 receptors with a neutralizing antibody reduced organoid number and corresponding surface area, potentially attributing to a significant decrease in AT2 cells (Supplementary Fig. 2). FACS assays revealed that the decrease in both AT1 and AT2 cells in *Plau*^*−/−*^ organoids were partially restored by A6 peptides (Fig. 1f). These experiments demonstrate that the binding of A6 peptides to CD44 receptors mediates the regulation of AT2 fate by uPA.

To confirm these observations, we sorted CD44^+^ and CD44^−^ AT2 cells in vivo. Significantly more CD44^+^ cells were harvested from wt than in *Plau*^*−/−*^ mice (Fig. 1g). *Plau*^*−/−*^CD44^+^ cells formed lesser organoids than those in wt controls (112 ± 10 vs. 182 ± 12). However, there was no significant difference in the number of CD44^−^ AT2 organoids (wt 30 ± 1 and *Plau*^*−/−*^ 33 ± 2). FACS assays confirmed that AT1 and AT2 cells in CD44^+^*Plau*^*−/−*^ organoids were fewer than those in wt CD44^+^ organoids. However, a significant decrease was observed only in AT1 but not in AT2 cells of CD44^-^*Plau*^*−/−*^ organoids.

We uncover a novel uPA-A6-CD44^+^-ENaC cascade for the AT2-mediated re-alveologenesis (Fig. 1h). The regulation of ENaC activity in oocytes by uPA appears controversial.^3,4^ This could be due to the differences in uPA preparations. We tested both LMW and HMW recombinant highly pure two-chain uPA (tc-uPA) from different companies and showed the same activation on human ENaC activity.^5^ This activation was eliminated by mutating S195A (catalytic site mutant) and the mixtures of tc-uPA with PAI-1, a specific inhibitor. In contrast, Bohnert reported^4^ that urine extract composed of 0.1 mg uPA and 24 mg other excipients, (i.e., disodium phosphate dodecahydrate, sodium dihydrogen phosphate dihydrate, and human albumin) did not activate human ENaC activity. As we reported, single-chain uPA (sc-uPA) alone did not regulate ENaC activity in vitro.^5^ In the presence of plasmin(ogen), as reported by Bohnert and in vivo, sc-uPA will be cleaved by plasmin to produce enzymatically active tc-uPA to activate ENaC activity.^4^ Whether sc-uPA or tc-uPA exists in the urine extract, and if other predominant components regulate ENaC activity is unknown. We reported that overexpression of the *Scnn1d* gene, a pseudogene in mice, significantly improved the development of organoids.^1^ Increased ENaC activity leads to increased cytosolic Na^+^ content, depolarized membrane potential, expanded cell volume, and regulation of the signal pathways and biological processes in a Na^+^-dependent manner. The contribution of ENaC function to epithelial wound healing implies the role of ENaC activity in the AT2 lineage. uPA released from other cells may serve as paracrine to initiate the uPA-A6-CD44-ENaC cascade in AT2 cells. In summary, we provide new evidence for the role of the fibrinolytic niche in the repair of ARDS. Further experiments to uncover its roles in COVID-19 and other types of ARDS could prove beneficial.

## Supplementary information

Supplementary file-v4 clear

## Data Availability

The data sets used for the current study are available from the corresponding author upon reasonable request.
