# Supplementary material for: Fibrinolytic niche is required for alveolar type 2 cell-mediated alveologenesis via a uPA-A6-CD44+-ENaC signal cascade
Source: Signal Transduct Target Ther. 2021 Feb 27;6:97. doi: 10.1038/s41392-021-00511-9 (PMC7910758; doi:10.1038/s41392-021-00511-9)
Supplement: Supplementary file 1 — Supplementary file-v4 clear [file 41392_2021_511_MOESM1_ESM.docx]

**Fibrinolytic niche is required for alveolar type 2 cell-mediated alveologenesis via a uPA-A6-CD44^+^-ENaC signal cascade in acute lung injury**

Gibran Ali^1, &^, Mo Zhang^1, 2, &^, Runzhen Zhao^1, &^, Krishan G. Jain^1^, Jianjun Chang^1, 3^, Satoshi Komatsu^1^, Beiyun Zhou^6,7,8,9^, Jiurong Liang^10^, Michael A Matthay^4,5^, Hong-Long Ji^1, 11, *^

^1^Department of Cellular and Molecular Biology, University of Texas Health Science Center at Tyler, Tyler, TX, USA; ^2^Institute of Lung and Molecular Therapy, Xinxiang Medical University, Xinxiang, Henan, China; ^3^Institute of Health Sciences, China Medical University, Shenyang, Liaoning, China; ^4^Department of Medicine and Anesthesia, ^5^Cardiovascular Research Institute, University of California San Francisco, San Francisco, CA, USA; ^6^Division of Pulmonary, Critical Care and Sleep Medicine, ^7^Hastings Center for Pulmonary Research, ^8^Norris Comprehensive Cancer Center, ^9^Department of Medicine, Keck School of Medicine, University of Southern California, Los Angeles, CA, USA; ^10^Department of Medicine, Cedars-Sinai Medical Center, Los Angeles, CA, USA; ^11^Texas Lung Injury Institute, University of Texas Health Science Center at Tyler, Tyler, TX, USA.

^&^These authors equally contributed to the study.

*** Correspondence to**: [honglong.ji@uthct.edu](mailto:honglong.ji@uthct.edu)

**This PDF file includes:**

Materials and Methods

Supplementary Text

Figures. S1 to S2

**MATERIALS & METHODS**

**Cells, antibodies, reagents, and key materials**

Major critical reagents are listed in the Table 1. General chemicals and reagents were purchased from Sigma.

**Animal husbandry**

All mice purchased from Jackson Laboratory were maintained in a pathogen-free facility. A 12-h light/dark cycle and ad libitum supply for food and water were provided. Age, sex, and weight-matched (4-12 months) wild type (wt) and *Plau*^-/-^ mice were sacrificed for experiments as approved by the Institute of Animal Care and Use Committee of the University of Texas Health Science Center at Tyler.

**Influenza-induced lung injury and immunohistology**

Wt and *Plau*^-/-^ mice were infected intranasally with a dose of 3,660 pfu influenza virus (type A/PR8/34 H1N1, Charles River, USA) diluted in 50 μl PBS per anesthetized animal. Control animals were intranasally injected with the same amount of PBS. Both control and infected mice were euthanized 5 d post infection. The trachea was tied with a suture followed by opening the chest cavity and removing the lungs subsequently. The dissected lungs were fixed with 10% neutral buffered formalin v/v (Richard-Allan Scientific) for 72 h at room temperature. Lung tissues were then dehydrated with increasing ethanol grades, embedded in paraffin, and sectioned at a thickness of 7 μm. As reported previously, influenza caused lung injury is not evenly.[^1^](#_ENREF_1)^,^[^2^](#_ENREF_2) Thus, we selected the same lobes and fields per the criteria of acute lung injury score for each stage to compare wt and *Plau*^-/-^ mice.

**Visualization and quantification of AT2 cells in mouse lung tissues**

Lung sections from influenza-infected mice were deparaffinized and rehydrated in xylene and a series of ethanol at cumulating concentrations and in H_2_O for 5 min. To unmask antigens, tissue slides were incubated with 10 mM sodium citrate buffer (Thermo Scientific), pH 6.0, for 20 min at 95°C, cooled for another 20 min at room temperature, and then washed with PBS. After blocking for 1 h with 1% BSA and 4% normal goat serum, tissue sections were incubated with following antibodies: anti-proSP-C (1:500, EMD Millipore), anti-pdpn (1:300, ThermoFisher) for mouse lung tissues. After 3 times washing with PBS, secondary antibodies were applied: goat anti-rabbit IgG AF488, goat anti-hamster AF568, and goat anti-mouse AF568. Tissue sections were stained with DAPI and sealed. Images were captured using a Zeiss LSM 510 confocal microscope. Images were captured containing at least 7 - 8 individual 1μm optical Z-sections. Z-stacks were obtained from at least five randomly selected areas. All images were subsequently processed with ImageJ software. AT2 cells were counted using a cell counter plug-in for ImageJ software. Cells were counted in at least 5 different areas to obtain a total above 1,000 cells and then analyzed statistically.

**Mouse AT2 isolation**

Mouse AT2 cells were isolated from wt, *Plau*^-/-^, and *Sepine1^Tg^* strains of C57BL/6 animals (Jackson Laboratory, USA) as previously reported with modifications.[^3^](#_ENREF_3) Briefly, mice were euthanized and exsanguinated, followed by perfusing lungs with 10 - 20 mL DPBS until pink lungs turned to white. The trachea was cannulated with a 20G catheter to instill 1.5 - 2.0 mL dispase followed by 0.5 mL of 1% low melting point agarose. The lungs were dissected and incubated in 50 U/mL dispase solution for 45 min at room temperature. The lungs were gently teased in DMEM/F-12 + 0.01% DNase I and incubated for 10 min at room temperature. Cells were passed through a serial filtration (100, 40, 30, and 10 µm cell strainers) and centrifuged at 300 × g for 10 min at 4^o^C. Cells were resuspended in 10 mL medium (DMEM/F-12 + 10% FBS + P/S) supplemented with biotinylated antibodies, rat anti-mouse CD16/32 (0.65 µg/million cells), rat anti-mouse CD45 (1.5 µg/million cells), and rat anti-mouse Ter119 (5 μg) and incubated for 30 min at 37^o^C on an incubator shaker at 60 rpm. Resuspended cells were then incubated with pre-washed streptavidin-coated magnetic particles for 30 min at room temperature to remove undesired cells. Selected cells were then resuspended in 10 mL medium (DMEM/F-12 + 10% FBS + P/S) and incubated for 30 min at 37^o^C in a sterile Petri dish to allow residual fibroblasts to adhere to the bottom of the dish. Suspended cells were transferred in plates pre-coated with mouse IgG for 2 h in a 5% CO_2_ incubator to remove macrophages. Unattached cells were collected and centrifuged at 300 × g for 10 min at 4^o^C. Cell pellets were then resuspended in a complete mouse medium (CMM: DMEM/F-12 supplemented with 2 mM L-glutamine, 0.25% bovine serum albumin, 10 mM HEPES, 0.1 mM non-essential amino acids, 0.05% ITS, 100 μg/mL primocin, and 10% newborn calf serum). The viability of harvested AT2 cells was assessed by the trypan blue exclusion assay followed by cell counting for the yield.

The purity of isolated AT2 cells was confirmed by the Papanicolaou stain, immunofluorescent staining with anti-proSP-C antibody, and fluorescence-activated cell sorting (FACS) with anti-EpCAM antibody. 1) **PAP stain**: freshly isolated AT2 cells suspended in DPBS + 10% FBS (2 × 10^5^ cells/mL) were centrifuged at 600 rpm for 4 min by a Shandon CytoCentrifuge. Slides were air-dried overnight and stained using a modified PAP stain method.[^4^](#_ENREF_4) Briefly, slides were stained with hematoxylin for 3.5 min. After rinsing in dH_2_O, slides were incubated in lithium carbonate for 2 min and rinsed with dH_2_O again. Slides were dehydrated in serial ethanol dilutions, then in xylene: ethanol (1:1) for 30 s, and finally in 100% xylene for 60 s. Slides were mounted with the Permount mounting medium. Randomly selected images were captured from 6 independent experiments with an Olympus BX41 microscope (40 ×). AT2 cells with large nuclei and blue colored granules spread in the cytoplasm were counted and calculated for purity (%). 2) **Immunofluorescent stain**: cytospinned freshly isolated AT2 cells and cells cultured on coverslips for 48 h were incubated with rabbit anti-proSP-C (1:500) overnight at 4^o^C. Secondary antibody, either Alexa Flour 488 or 568-conjugated anti-rabbit IgG, was added to recognize proSP-C antibody. 3) **FACS**: Briefly, cells were resuspended in staining buffer containing 0.1 mM EDTA. Cells were incubated with Alexa Fluor 488 conjugated anti-mouse CD326 for 30 min on ice in the dark. Cells were washed and resuspended in staining buffer and analyzed for antibody expression on BD FACSCalibur^TM^. Data were analyzed with FlowJo 10.1 software. The purity of the final cell suspensions was about 93% (**Fig. S1a-c**).

**CD44^+^ AT2 cells sorting and analysis by FACS**

Freshly isolated cells were seeded on either collagen IV (for mouse AT2, 10 μg/cm^2^) coated plates for 24 - 36 h to revive CD44 expression diminished by digestive enzymes. Both unattached and attached (trypsinized) cells were collected and blocked with 1% BSA, 4% normal goat serum in PBS. Cells were stained with AF488-EpCAM (BioLegend), and their respective isotypes. Cells were sorted using a Beckman Coulter MoFlo high-speed cell sorter. Unstained, isotype and single-color controls were performed. The gates for CD44 and EpCAM were set based on the results of isotype, and single-color controls were run in parallel. The results were analyzed using FlowJo 10.1 software.

**3D polarized AT2 monolayers**

Transwell inserts (Costar 3470: 0.4 μm pore size, 0.33 cm^2^ area; Corning Costar, USA) were pre-coated with mouse laminin 1 at 10 μg/cm^2^ (for mouse AT2 cells; Trevigen, USA) for 4 - 6 h at 37^o^C or with rat tail collagen I at 10 μg/cm^2^ for 1 h at 37^o^C. Freshly isolated AT2 cells were seeded at 10^6^ cells/cm^2^. The CMM medium (600 μL) was added to the basolateral side of each transwell. The culture medium on the basolateral side was replaced with a serum-free medium in 48 h post seeding. Transepithelial resistance (R_T_, Ω) and potential difference (V_T_, mV) were measured using an epithelial voltohmmeter (EVOM: World Precision Instrument, USA) in 72 h. The culture medium was then replaced with serum-free media and maintained with the air-liquid interface until day 5. Monolayers were maintained in a humidified 5% CO_2_ air incubator at 37°C.

**Measurements of bioelectric properties in AT2 monolayers**

Transepithelial short-circuit current (I_SC_, μA/cm^2^) in AT2 monolayers was measured with an 8-channel voltage-clamp amplifier (Physiological instruments, USA) as previously described.[^5^](#_ENREF_5) Briefly, AT2 monolayers were mounted in the vertical Ussing Chambers bathed with solutions containing (in mM): 120 NaCl, 25 NaHCO_3_, 3.3 KH_2_PO_4_, 0.83 K_2_HPO_4_, 1.2 CaCl_2_, 1.2 MgCl_2_, 10 HEPES, 10 mannitol (apical compartment), or 10 D-glucose (basolateral compartment). Each solution was iso-osmotic. The transwell cultures were bubbled continuously with a gas mixture of 95% O_2_-5% CO_2_. The transmonolayer potential was short-circuited to 0 mV, and a 10-mV pulse of 1-s duration was imposed every 10 s to monitor transepithelial resistance. Data were collected with the Acquire and Analyze program (version 2.3; Physiologic Instruments). When the Isc level reached a plateau, compounds were pipetted to the apical compartment. Our results were supported by that ~20 % of apical conductance was inhibited by amiloride in rat fetal alveolar epithelial monolayers.[^6^](#_ENREF_6) To the best of our knowledge, we computed the IC_50_ value of amiloride in mouse AT2 monolayers for the first time. The lesser sensitivity to amiloride could be due to holding potential and the polarization of monolayers, as that ENaC could function as a amiloride-resistant, non-selective cation channels in unpolarized cells[^7^](#_ENREF_7)^,^[^8^](#_ENREF_8) and that the affinity of amiloride to ENaC binding site was voltage dependent.[^9^](#_ENREF_9)

**3D organotypic cultures of AT2 cells**

AT2 cells were cultured as organoids as previously described.[^10^](#_ENREF_10) Briefly, Mlg-2908 cells (2 × 10^5^ cells/mL) were mixed with 6,000 primary AT2 cells and pelleted down for each transwell. Cells were then resuspended into a 100 µL mixture (1:1) of growth factor reduced matrigel (Corning, USA) and organoid medium (DMEM/F12 supplemented with 2 mM L-glutamine, 10 % active FBS, 1% ITS, and 10 μM ALK inhibitor). On each 0.33 cm^2^ insert (Corning Costar, USA) 50 uL mixed cells were seeded and incubated at 37°C for 30 min to allow the matrix to solidify. Then 410 µL culture medium was added to the bottom well and changed half (200 µL) of the medium every other day. The DIC images of organoids (diameter ≥ 50 μm) were visualized with an Olympus IX 73 microscope (4× objective, Olympus, Japan) with an Hamamatsu photonics CMOS camera (Orca Flash 4.0; 2,048 × 2,048 pixels) on a designed day post seeding. The surface area of individual organoid was measured with Image J. The sum of all organoids on each transwell insert was the total surface area. Histologic images of organoids were captured from sliced matrigel. Matrigel containing organoids was fixed with 4% paraformaldehyde in PBS for 1 h, dehydrated with increasing ethanol grades, and embedded in paraffin blocks. Sections at the thickness of 7 μm were fixed for an additional 5 min in 4% paraformaldehyde, then H & E stain was performed as described previously.[^11^](#_ENREF_11)

**Quantification of AT1 and AT2 cells in organoids and monolayers**

Anti-pdpn and anti-sftpc antibodies were used to detect AT1 and AT2 cells, respectively. Fluorescence conjugated secondary antibodies, goat anti-hamster AF568 and goat anti-rabbit IgG AF488 were used. Fluorescent images were projected with a Zeiss LSM 510 confocal microscope and stacked with a Fiji plug-in for ImageJ. Monolayers and organoids were scanned for Z sections with optimal depth from top to bottom. Images were stacked for pdpn and sftpc signal separately to count the number of positive cells precisely with a cell counter plug-in of ImageJ. Alternatively, cells were collected from organoids and monolayers and sorted by FACS. Each slide was scanned for at least 6 different fields (n = 3 animals/experiments). For 3D organoids, all Z sections were stacked from top to bottom and saved as .avi files.

**EdU assay for DNA synthesis**

AT2 cells with active DNA synthesis in organoids and monolayers were detected with a Click-iT^TM^ EdU assay kit. Organoids and monolayers from both wt and *Plau*^-/-^ groups were stained with the Click-iT^TM^ EdU Alexa flour 488 following the manufacturer’s instructions. Images were captured and analyzed for the percentage of EdU^+^ cells in different experimental groups. Ten randomly selected images across the monolayer from 3 independent experiments were captured and counted for total cells and EdU^+^ portion. For organotypic cultures, all 3 organoid types were scanned (n = 5 colonies for each type). The z sections were stacked separately for DAPI (blue) and EdU (green) to produce a 3D structure of organoids for counting total and EdU^+^ cells, respectively, using a cell counter plug-in for the ImageJ. The percentage of EdU^+^ cells was calculated for each group, and the difference among groups was compared statistically.

**Application of A6 peptide and CD44-blocking antibody to organoids**

A6 and scrambled A6 (sA6) peptides were synthesized by Genscript. Stock solutions (2 mM) were prepared by dissolving peptides in water according to the manufacturer’s instruction. For 3D matrigel cultures, sorted *Plau*^-/-^ AT2 cells were preincubated with either A6 or sA6 peptide (1 μM) for 30 min at room temperature. Wt AT2 cells were treated with 30 ng/mL CD44-blocking antibody for 30 min at room temperature. The same concentrations of A6, sA6 peptides, and CD44-blocking antibody were added to both the matrigel/medium mix and the culture medium placed under the transwell inserts. The culture medium was changed every 48 h.

**Organoid dissociation and FACS**

For analysis of AT2 cell proliferation and differentiation, AT2 organoids from different experimental groups were isolated from matrigel with dispase (10 U/mL) and dissociated in 0.25% trypsin-EDTA to get a single-cell suspension. Cells were then stained with antibodies AF488 conjugated EpCAM, APC conjugated ICAM, and APC conjugated PDPN. Gates for both colors were set by unstained cells and isotype controls for each antibody. We have used a double color staining strategy to enhance the separation of AT1 and AT2 cells in the dissociated cells from organoids. Cells were analyzed by FACSCaliber^TM^ (BD, USA), and the results were analyzed using FlowJow 10.1 software.

**Statistical analysis**

Data were presented as mean ± s.e.m. No animals were excluded. Normality tests were performed to determine whether the data were parametric or not. If the data were normally distributed and the variance between groups was not significantly different, mean differences in measured variables between the experimental and control group were assessed with the Student’s two-tailed t-tests or one-way ANOVA followed by the Tukey’s or Bonferroni’s post hoc test. Otherwise, the Mann-Whitney U test was applied for analyzing non-parametric results. Two-way ANOVA followed by Sidak’s multiple-comparison test was used for multiple comparisons. Meanwhile, the actual power of the sample size was analyzed. Mean differences were considered statistically significant at the levels of P < 0.05, P < 0.01 and P < 0.001. Origin Pro 2018 was used for statistical analysis and plotting.

**Figure Legends:**

**Figure S1**. Comparison of AT2 organoids between wt and *Plau^-/-^* groups 12 days post planting in 3D matrigel. **a** DIC images. Phase-contrast images were captured (4 ×). Scale bar, 1 mm. **b** Colony-forming efficiency (CFE, left), counting (middle), and surface area (right). Colonies with a diameter of < 50 μm were not counted. *** P < 0.001 and **P < 0.01 vs controls. n = 7.

**Figure S2.** Effects of A6 peptide and CD44 blocking antibody on the formation of AT2 organoids. **a** Representative DIC images of AT2 organoids. From left to right, wt control organoids (WT), wt organoids treated with CD44 blocking antibody (CD44 Ab), *Plau*^-/-^ AT2 organoids (*Plau^-/-^*), and *Plau*^-/-^ organoids treated with A6 peptide (A6). 4 ×. **b** Organoid number. n = 6 replicates per experiment, n = 6 mice/genotype. * P < 0.05 and **P < 0.01 vs controls. **c** Surface area of organoids. * P < 0.05 and **P < 0.01 vs controls. n=6. Data in **b** & **c** were mean ±sem and analyzed by Student’s t-test.

**Figure S1**.


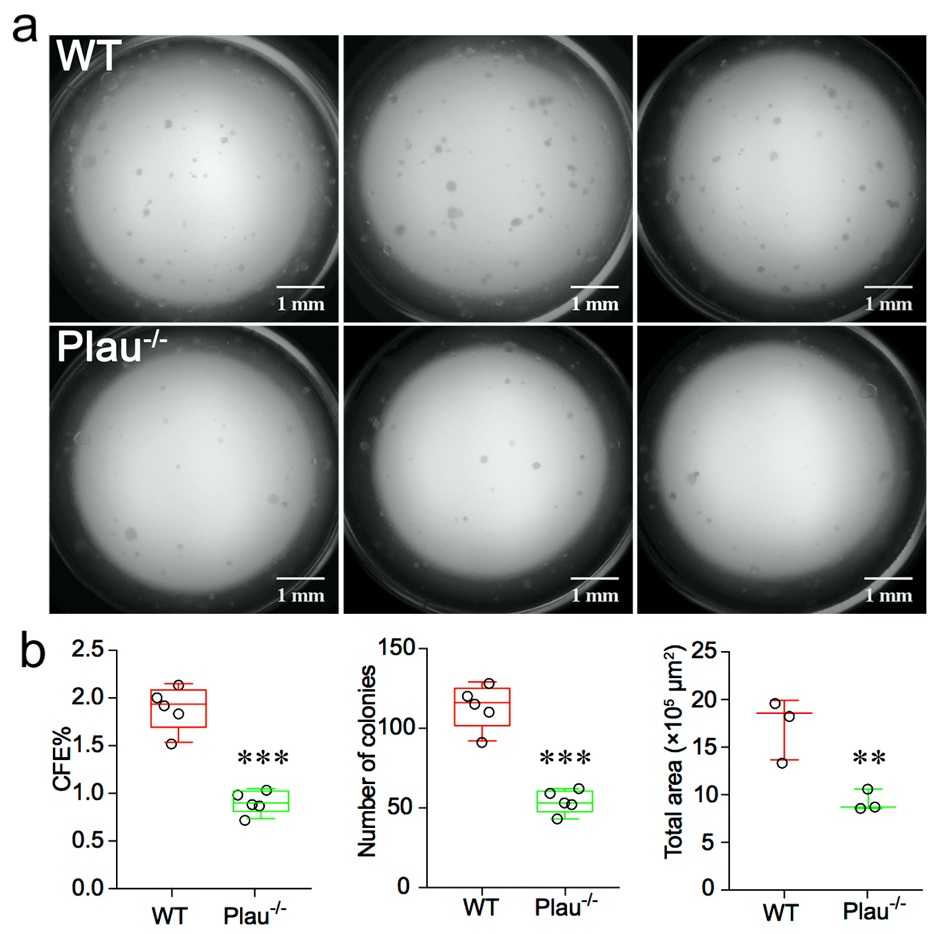


**Figure S2**.


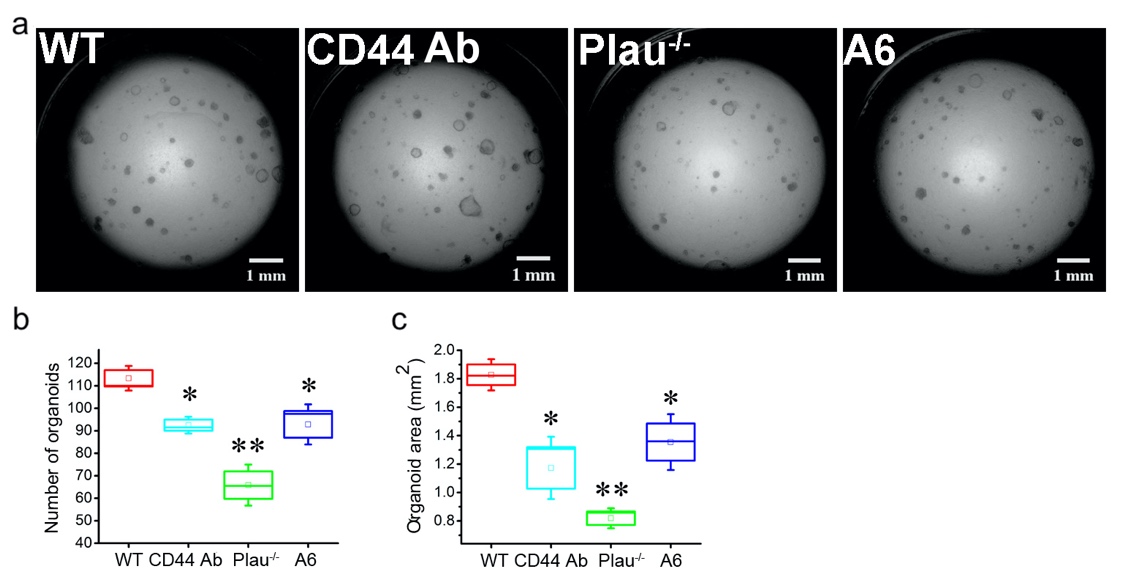


**References**

1 Ong, J. W. J. *et al.* Insights into early recovery from influenza pneumonia by spatial and temporal quantification of putative lung regenerating cells and by lung proteomics. *Cells* **8**, 975 (2019).

2 Traylor, Z. P., Aeffner, F. & Davis, I. C. Influenza A H1N1 induces declines in alveolar gas exchange in mice consistent with rapid post-infection progression from acute lung injury to ARDS. *Influenza Other Respir Viruses* **7**, 472-479 (2013).

3 Demaio, L. *et al.* Characterization of mouse alveolar epithelial cell monolayers. *Am J Physiol Lung Cell Mol Physiol* **296**, L1051-1058 (2009).

4 Dobbs, L. G. Isolation and culture of alveolar type II cells. *Am J Physiol* **258**, L134-147 (1990).

5 Demaio L, T. W., Balverde Z., Alvarez, J.R., Kim, K.J., Kelley, D.G., Senior, R.M., Crandall, E.D., Borok, Z. Characterization of mouse alveolar epithelial cell monolayers. *Am J Physiol Lung Cell Mol Physiol* **296**, L1051-L1058 (2009).

6 O'Brodovich, H., Rafii, B. & Post, M. Bioelectric properties of fetal alveolar epithelial monolayers. *Am J Physiol* **258**, L201-206 (1990).

7 Yue, G. *et al.* Culture-induced alterations in alveolar type II cell Na^+^ conductance. *Am J Physiol* **265**, C630-640 (1993).

8 Jain, L., Chen, X. J., Ramosevac, S., Brown, L. A. & Eaton, D. C. Expression of highly selective sodium channels in alveolar type II cells is determined by culture conditions. *Am J Physiol Lung Cell Mol Physiol* **280**, L646-658 (2001).

9 Ji, H. L. *et al.* Delta-subunit confers novel biophysical features to alpha beta gamma-human epithelial sodium channel (ENaC) via a physical interaction. *J Biol Chem* **281**, 8233-8241 (2006).

10 Liang, J. *et al.* Hyaluronan and TLR4 promote surfactant-protein-C-positive alveolar progenitor cell renewal and prevent severe pulmonary fibrosis in mice. *Nat Med* **22**, 1285-1293 (2016).

11 Kaisani, A. *et al.* Branching morphogenesis of immortalized human bronchial epithelial cells in three-dimensional culture. *Differentiation* **87**, 119-126 (2014).
